# A Cadaveric Study on the Course of the Cervical Segment of the Internal Carotid Artery and Its Variations

**DOI:** 10.7759/cureus.7663

**Published:** 2020-04-13

**Authors:** Sangeetha Arumugam, Nandha Kumar Subbiah

**Affiliations:** 1 Anatomy, Katuri Medical College and Hospital, Guntur, IND; 2 Anatomy, All India Institute of Medical Sciences, Mangalagiri, IND

**Keywords:** carotid variations, carotid bifurcations, kinking, coiling

## Abstract

Introduction

The internal carotid artery is the chief source of blood supply to the brain. Variations of the internal carotid artery, such as curved, kinking, and coiling, might result in significant neurovascular problems due to alterations in the dynamics of blood flow. The elongated and tortuous course of the internal carotid artery in the cervical region has a high chance of being damaged during head and neck surgeries.

Method

The study aims to observe the course and variations of the internal carotid artery in 50 cadaveric hemi-neck specimens of both sexes. The internal carotid artery was traced from its origin until its termination in the base of the skull. Variations in the course, position, and the level of the carotid bifurcation were observed and analysed.

Result

Out of the 50 specimens, a higher carotid bifurcation was observed in 40% of the specimens and 28% showed variations in the course, such as curved (18%), kinking (8%), and coiling (2%). The position of the internal carotid artery was reversed in 16% of the specimens.

Conclusion

Curved, kinking, and coiling types of carotid artery variations observed in the present study had right-sided and female predominance. Knowledge about such variations is essential for the diagnosis and management of neurological disorders and in head and neck surgeries.

## Introduction

Carotid arteries are the main arteries of the head and neck region [1]. The common carotid artery bifurcates into the internal and external carotid artery (ECA). The ECA supplies the face, scalp, and cervical viscera. The internal carotid artery (ICA) supplies the intracranial structures, orbit, and scalp [2]. The ICA consists of four parts from its origin to its termination; cervical, petrous, cavernous, and cerebral parts [1, 3]. Anatomical variations of the carotid arteries could be secondary to embryological malformations [4-5]. Standard textbooks and cadaveric studies have seldom mentioned carotid variations. However, they commonly appear in carotid angiographic (CA) studies where the cervical part of the ICA is shown to have unusual forms of coiling and kinking [6-7]. Variations in the course of the ICA lead to alteration in blood flow dynamics, resulting in various neurological manifestations [8-9]. Outcomes of invasive procedures, such as carotid stenting, endarterectomy, and head/neck surgeries, may be significantly affected by a deviation in the normal course of the ICA [10-11]. The type of variations and their levels of occurrence are clinically significant. Cadaveric studies on variations of the ICA, its course, and subtypes are scanty. Hence, the present study aims to record the variations in the course of ICA and its subtypes in human cadavers.

## Materials and methods

**Figure 1:**
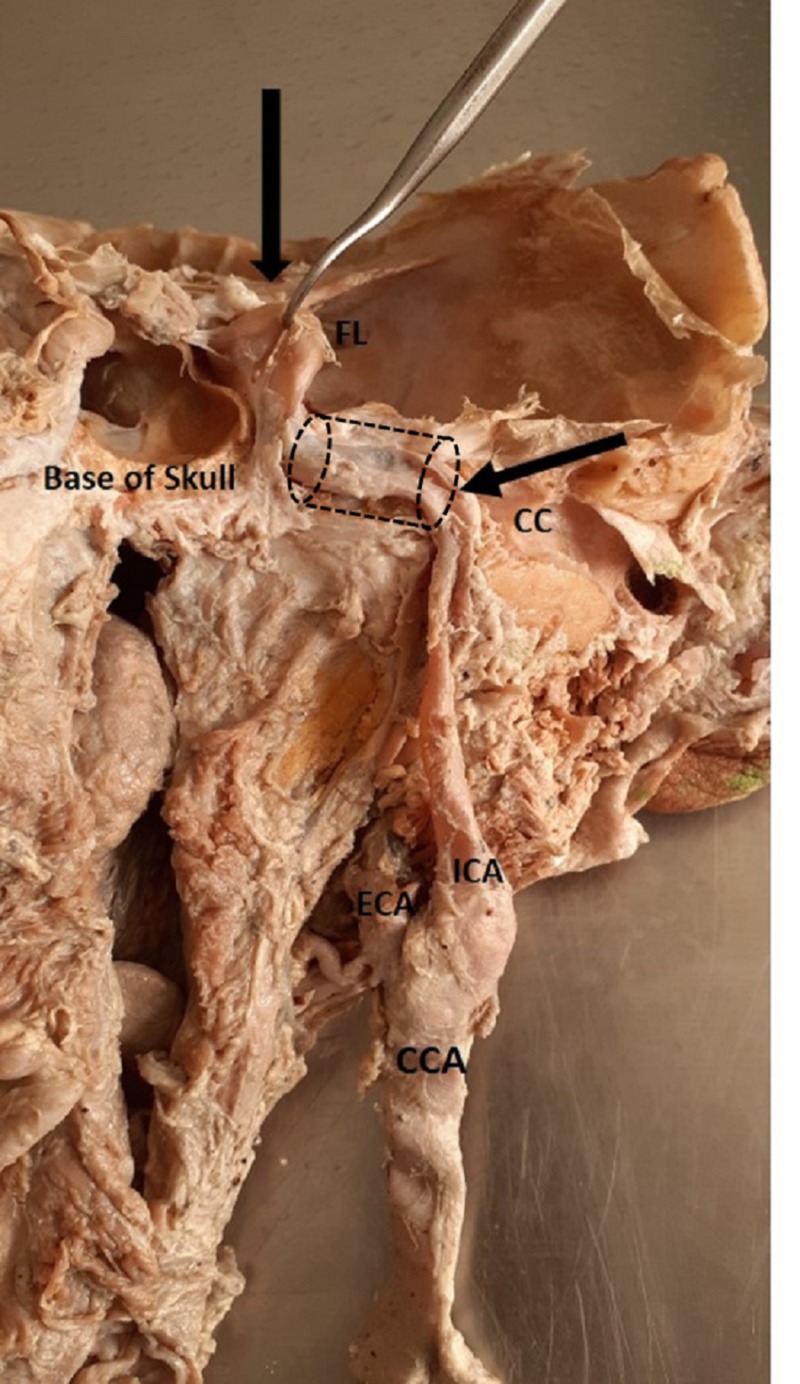
Posterior view of the normal course of the internal carotid artery (ICA) from its origin to its entry into the carotid canal (CC) at the base of the skull CCA: common carotid artery; ECA: external carotid artery; FL: foramen lacerum

The present study was conducted in the Department of Anatomy, Katuri Medical College and Hospital, India after obtaining clearance from the institute's ethical clearance committee. Fifty hemi-neck specimens collected from formalin-fixed embalmed cadavers of the South Indian population (males - 20 and females - 5) were dissected. Each ICA was traced from its origin to its termination to the carotid canal at the base of the skull (Figure 1). The age of the cadavers was between 50 to 70 years. Variations in the course, position, and the level of the carotid bifurcation were noted. Similarly, deviations in the course of the ICA and its subtypes were documented and photographed. The data obtained were tabulated and subjected to statistical analysis. 

## Results

Variations in the level of the carotid bifurcation

**Figure 2:**
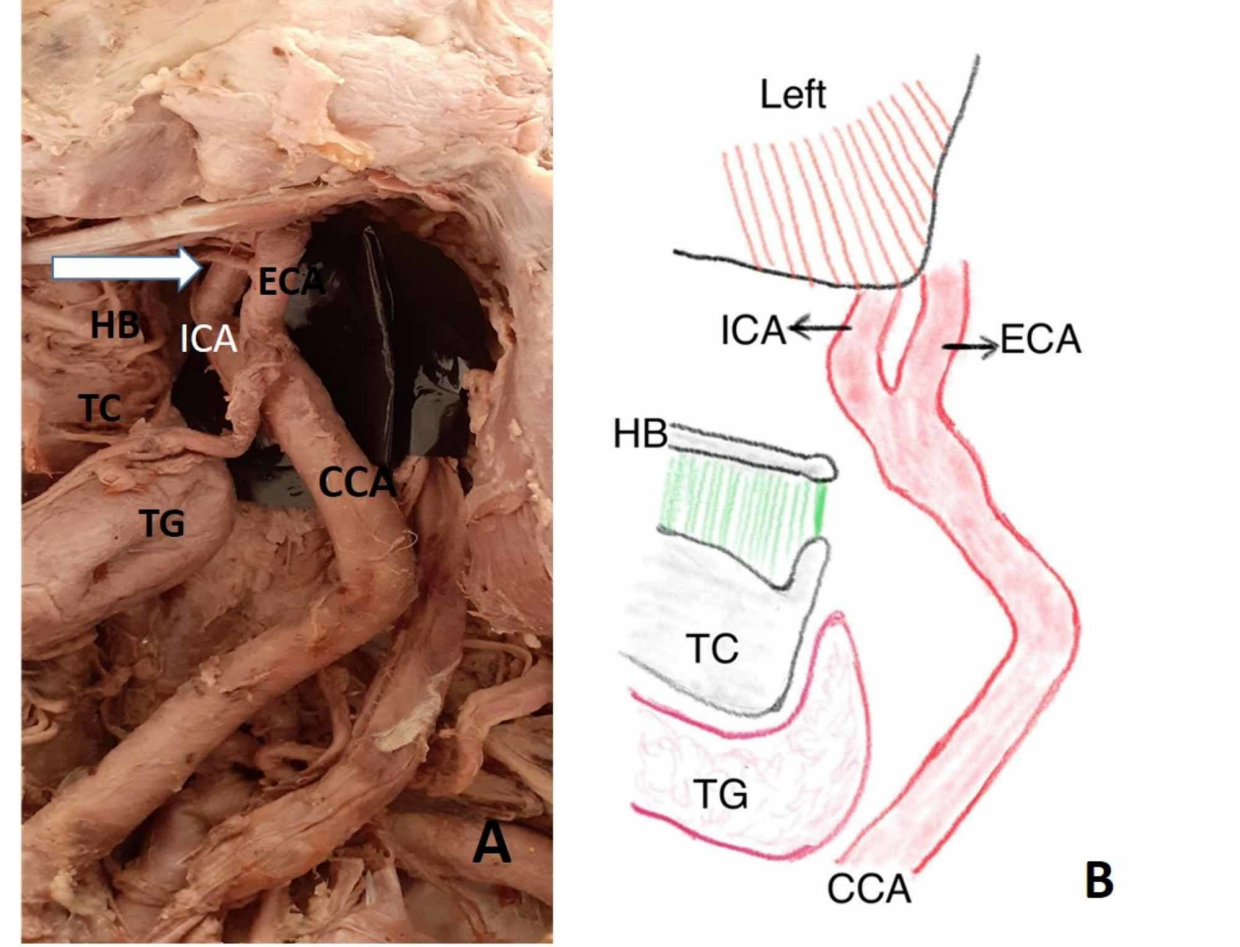
High carotid bifurcation with reversal of ICA and ECA positions A) Left hemi-neck specimen showing the carotid bifurcation (CB) above the hyoid bone with medial ICA and lateral ECA; B) schematic diagram showing the variations CCA: common carotid artery; ECA: external carotid artery; HB: hyoid bone; ICA: internal carotid artery; TC: thyroid cartilage; TG: thyroid gland

The level of the carotid bifurcation (CB) was categorized into three levels: normal - at the upper border of the thyroid cartilage (TC), high - above the TC at the level of the hyoid bone (HB) or between TC and HB, and low - below the upper border of the TC. Out of the 50 hemi-neck specimens, 30 (60%) specimens had a normal CB at the upper border of the TC and 20 (40%) specimens had a high CB, of which 12 (24%) were noted at the level of the HB (Figure 2) and eight (16%) specimens were just below the angle of the mandible (Table 1). None of the specimens had a low CB level. 

**Table 1:** Levels of the Carotid Bifurcation

Levels of the Carotid Bifurcation	Male	Female	Total / Percentage
Upper border of the thyroid cartilage	25	5	30 (60%)
At the level of the hyoid bone	2	10	12 (24%)
Above the level of the hyoid bone	2	6	8 (16%)
	29	21	50

Variations in the positions of the internal and external carotid arteries

**Figure 3:**
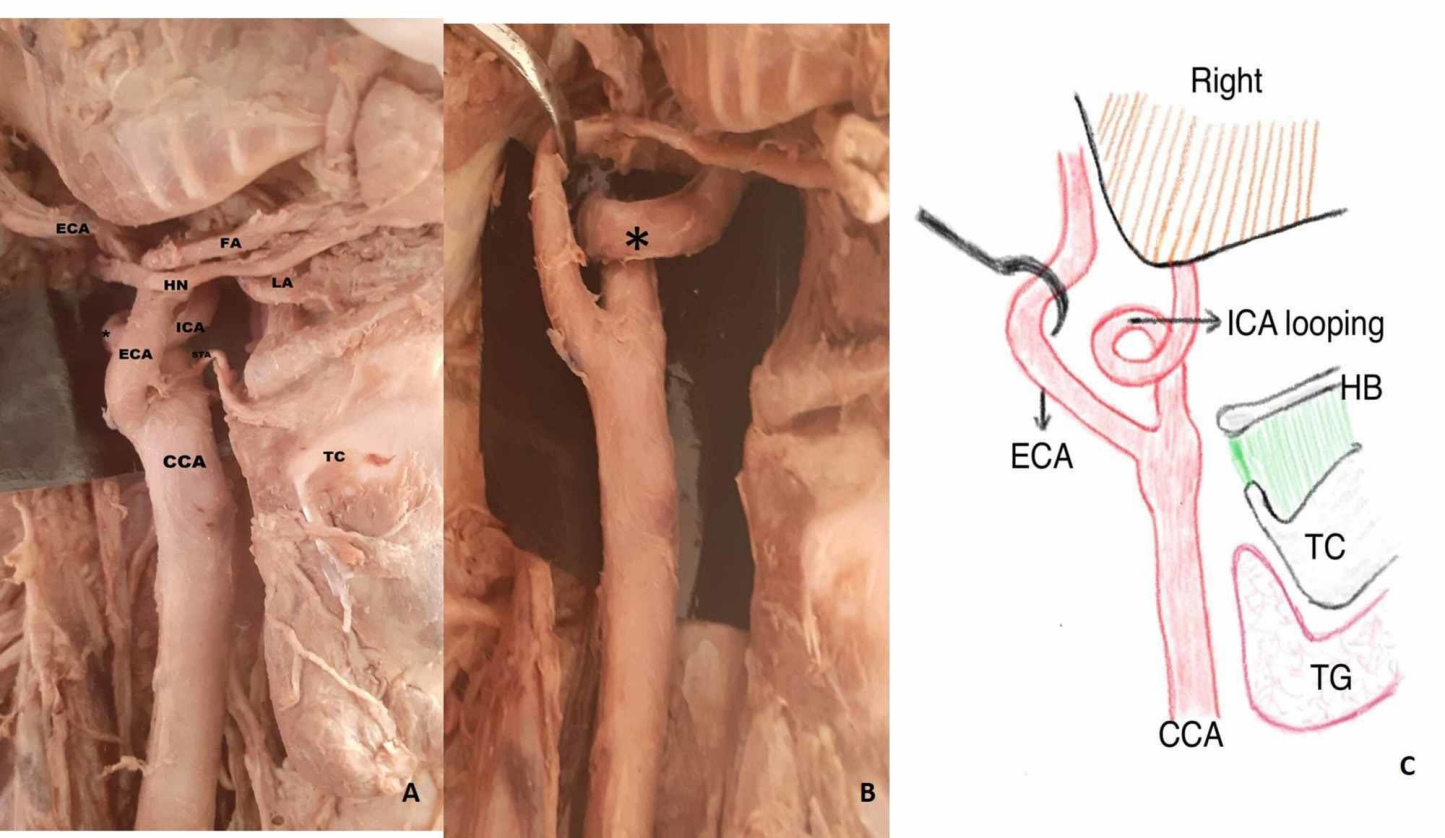
High carotid bifurcation and coiling of ICA (Type 4) A) CCA bifurcation at the level of the HB into the lateral ECA and medial ICA; branches of the ECA - superior thyroid artery (STA), lingual artery (LA), and facial artery (FA) - crossing over the ICA from lateral to medial side; B) after cutting the branches of the ECA, coiling of the ICA is seen at the level of the HB posteromedial to lateral ECA; C) schematic diagram showing the variations CCA: common carotid artery; ECA: external carotid artery; HB: hyoid bone; ICA: internal carotid artery

Normally, the ECA is anteromedial in position and the ICA is anterolateral after the CB. In the present study, a reversal of the said position was observed in eight out of 50 specimens. Among them, the ICA originated on the medial side and coursed medially up to the carotid canal (CC) situated at the base of the skull. The laterally placed ECA and its anterior branches (superior thyroid, lingual, and facial arteries) crossed the ICA from the lateral to the medial side as shown in Figures 2A, 3A-C. In all the eight specimens, the CB was seen at a higher level. In six specimens, it was present on the right hemi-neck and two on the left side.

Variations in the course of the internal carotid artery

**Figure 4:**
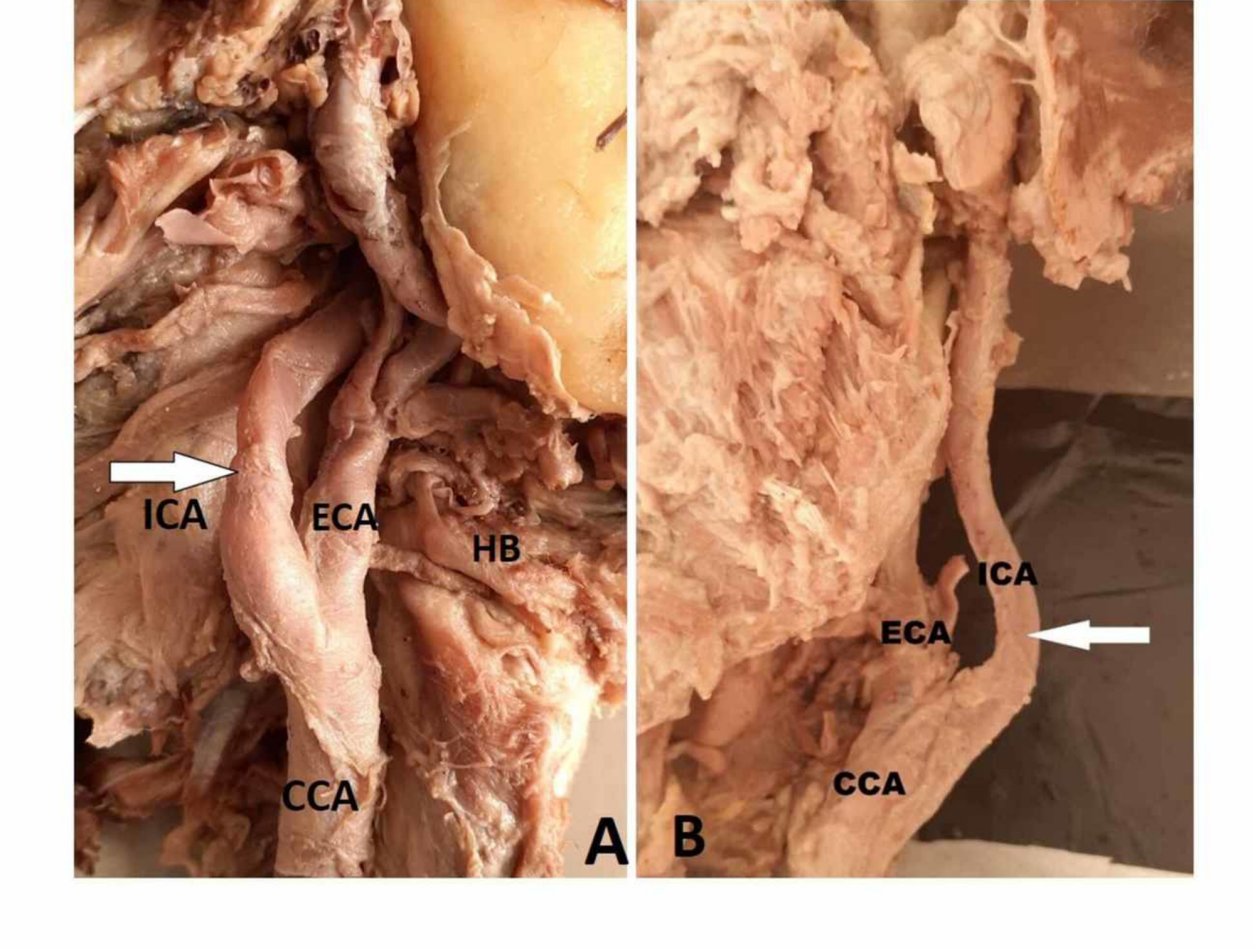
Curved course of the ICA (Type 2) A) Right hemi-neck specimen showing the CB at the hyoid bone with a Type 2 curved course of the ICA; B) left hemi-neck specimen showing a curved ICA CB: carotid bifurcation; CCA: common carotid artery; ECA: external carotid artery; HB: hyoid bone; ICA: internal carotid artery

**Figure 5:**
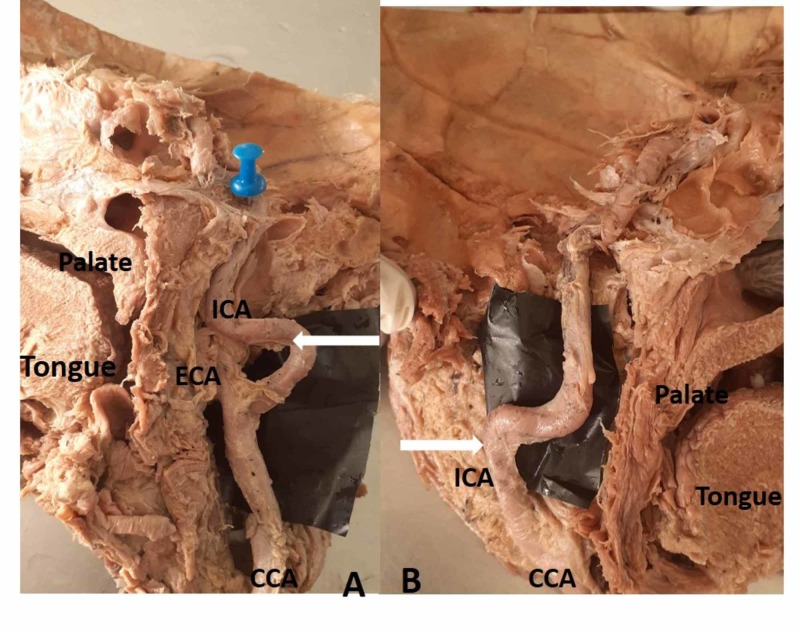
Kinking of the ICA (Type 3) A) Medial view of a right hemi-neck specimen showing Type 3 kinking of the ICA at the level of the oropharynx; B) medial view of the left hemi-neck specimen showing kinking CCA: common carotid artery; ECA: external carotid artery; ICA: internal carotid artery

The ICA at its origin lies lateral and ECA lies medial, but ECA courses backward and laterally into the parotid gland and terminates into the maxillary artery and superficial temporal artery. The ICA runs laterally and its course is straight from its origin up to the carotid canal in the neck as shown in (Figure 1). However, we observed a deviation in the course of the ICA at the lower and upper cervical region. Based on the ICA course, it was divided into four types: Type 1 - straight (Figure 1), Type 2 - curved (Figure 4A-B), Type 3 - kinked (Figure 5A-B), and Type 4 - coiling/looping (Figure 3A-C, Table 2). Out of the 50 hemi-neck specimens, Type 1 was seen in 36 specimens (72%) and Type 2 was seen in nine specimens where the direction of the curvature was seen both on the medial and lateral side (Figure 4A-B). Type 3 was seen in four specimens where it was kinked at various levels from its origin up to the carotid canal (Figure 5A). Kinking was right-angled in one specimen at the level of the palate (Figure 5B). In other specimens, it was kinked to a lesser degree of angulation at its origin. Type 4 was seen in one specimen (2%) just above the level of the hyoid bone on the right side (Figure 3A-C). After bifurcation of the CCA at the level of the hyoid bone, branches of the ECA crossed the ICA from lateral to medial to supply the thyroid gland, tongue, and face, respectively (Figure 3A). After cutting and reflecting these branches, the ICA showed a complete coiling posteromedial to the ECA (Figure 3B). A simplified schematic diagram of the variation is shown in Figure 3C.

**Table 2:** Types of Internal Carold Artery (ICA) Variations

Types of ICA variations	Number of Specimens	Percentage
Type 1 - Straight	36	72%
Type 2 - Curved	9	18%
Type 3 - Kinking	4	8%
Type 4 - Coiling	1	2%

## Discussion

The present study is aimed at studying the variation in the course of the ICA in the neck region. It is essential to know the deviation in its course because of the relation to the pharyngeal wall and its proximity to various other neurovascular structures. The ICA is the chief source of blood supply to intracranial structures through its contribution to the circle of Willis. The ICA does not supply any branches in the neck region. In normal anatomy, the CCA bifurcates into the ICA and ECA at the level of the upper border of the thyroid cartilage. The ICA lies anterolateral and the ECA lies anteromedially [1-2]. The level of the carotid bifurcation is taken as the point of the ECA origin. Embryologically, the proximal part of the third aortic arch forms the CCA and the distal portion of the third aortic arch forms the ICA. The ECA arises as a sprout from the third arch artery medially [3-4]. Based on the point of origin of the ECA, the level of the CB differs. The level of the CB is clinically important to identify the branches of the ECA for ligation of arteries during head and neck surgeries [11-12]. Radiologically, it is crucial for ICA stenting and other procedures. In normal anatomy, the CB takes place at the upper border of the thyroid cartilage, corresponding to the cervical vertebral level C4-C5.

The common carotid artery bifurcates at the level of the upper border of the thyroid cartilage (C4-C5). Earlier studies have reported a higher level of the CB more frequently compared to the low level of the CB [13-14]. In the present study, 20 specimens (40%) showed the CB above the normal level. Mompeo and Bajo reported a similar value of 36.85% of high CB samples [15]. Out of 20 specimens in the present study, eight had the CB just below the angle of the mandible. A CB below the angle of the mandible lies close to the hypoglossal nerve which may be damaged during superior thyroid artery ligation [16]. A high CB with reversal of the position of the ICA and ECA was seen in eight specimens (six on the right side and two on the left side, mostly in female cadavers). Reversal of position of vessels (lateral ECA and medial ICA) can be explained as a rotation of the common carotid artery secondary to its elongation and tortuosity [17-18]. Due to the lateral shift of the external carotid artery, its anterior branches cross over the ICA to supply thyroid, tongue, and face. The incidence of lateral ECA is very rarely reported in cadavers. Bussaka et al. reported the lateral position of the ECA in 17 cases (4.3%), of which 13 cases were on the right side and four cases were on the left [18]. The values of the present study also show a higher incidence of lateral ECA on the right side compared to the left. Further, angiographic and ultrasound studies showed the incidence of a lateral ECA as 4% to 12.3% and most often on the right-side [19-20]. So far, only a few case reports have reported a lateral ECA in cadavers [21-22]. It is essential to know the exact anatomical position of the ECA. In the case of ECA ligation for surgeries, chances of ligation of the ICA is higher due to the change in the normal position of the vessels. Hence, in any procedures involving the CCA, ECA, and ICA, carotid angiography should be done prior to any interventions.

The ICA course is divided into four types based on the Weibel and Fields classification [9]: Type 1 - straight, Type 2 - curved (S or C elongation) with medial or lateral deviation of the affected segment, Type 3 - kinking, and Type 4 - coiling or loop formation. The present study showed Type 1 in 72% of specimens, Type 2 in 18%, Type 3 in 8%, and Type 4 in 2% (Table 2). In carcinoma of the buccal mucosa and larynx, neck dissection surgeries are done at the level of the hyoid bone. The presence of an S/C curved ICA at that level stands a high chance to be injured, leading to fatal complications [23-24]. In four specimens, kinking was seen at the level of the oropharynx, which was medially deviated. This type of variation is may be injured during tonsillectomy and other pharyngeal surgeries. Therefore, it is essential to study the distance and course of the ICA concerning palatine tonsils [23]. The etiology for kinking of the vessels could be associated with arteriosclerosis, stenosis, vasculitis, atrophic dilatation, or loss of elasticity of the vessel wall [9]. A similar study done on the course of femoral and vertebral vessels showed that increased curving and kinking is associated with increasing age [25]. In the present study, since all these specimens were from elderly individuals (50 - 70 yrs), the reason for curving and kinking could be due to loss of elasticity of the vessel wall. An alteration in the fluid dynamics in a kinked vessel causing degenerative changes in the vessel wall and leading to kinking has also been reported [8-9].

An elongation of the ICA in a restricted space causes tortuosity, resulting in coiling/loop formation (Type 4) in a circular pattern. Embryologically, during the formation of the ICA, an arterial loop connects the third arch artery and dorsal aorta. This loop disappears as the developing heart and blood vessels descend into the thorax. Persistence of this loop may form the coiling of the ICA [4-5]. In the current study, coiling was seen in an elderly female cadaver at the level of hyoid bone as shown in Figure 3A-C. It could be either congenital or senile changes of the vessel wall. According to studies on carotid angiography, coiling occurs in 5% to 15% of patients in an unselected angiographic series due to atherosclerosis [9]. In the present case, being an elderly individual, coiling may have been secondary to atherosclerotic changes of the vessel wall. Similar findings have been reported by Ovchinnikov et al. and Nirmala and Sharieff, with unilateral elongation and coiling of the ICA in an elderly cadaver [24, 26]. However, the unique feature of the present case is the higher CB with the reverse position of the ECA and ICA and the presence of coiling. Curving and coiling have a higher chance of rupture compared to kinking [23]. Hence, knowledge about the level of coiling is important for clinicians to prevent injury of the vessel during radiological and surgical procedures of the neck.

## Conclusions

The present study is one among the few cadaver-based reports outlining the variation in the course and termination levels of the ICA. Our findings show four types of the ICA course; straight (72%), curved (18%), kinking (8%), and coiling (2%). Also, we report an increased incidence of high carotid bifurcations, with the reversal in position of ICA and ECA. The reversal was seen more often on the right side and in female cadavers. The described ICA variations are of clinical relevance in the context of neck surgeries and other routine treatment procedures involving the neck region. Hence, radiologists and surgeons should be aware of such dolichoarteriopathies before surgery, and the same must be identified by imaging methods to prevent iatrogenic complications. 
